# Shelf life of ground beef enriched with omega‐3 and/or conjugated linoleic acid and use of grape seed extract to inhibit lipid oxidation

**DOI:** 10.1002/fsn3.251

**Published:** 2015-07-29

**Authors:** Inmaculada Gómez, María J. Beriain, Jose A. Mendizabal, Carolina Realini, Antonio Purroy

**Affiliations:** ^1^ E.T.S. Ingenieros AgrónomosUniversidad Pública de NavarraCampus de ArrosadíaPamplona31006Spain; ^2^ Centro IRTAFinca Camps i ArnetMonells17121Spain

**Keywords:** Conjugated linoleic acid, grape seed extract, ground beef, omega‐3, shelf life

## Abstract

The shelf life and oxidative stability of refrigerated raw ground beef enriched with omega‐3 and/or conjugated linoleic acid (CLA) were studied. Grape seed extract (GSE) was used to inhibit lipid oxidation in the ground beef. Eight treatments of ground beef were established according to the enrichment of beef (control, enriched with omega‐3, with CLA, or with omega‐3 plus CLA) and the use of GSE (0 and 250 mg GSE/kg product). Fresh beef was ground and mixed with GSE and salt. Treatments of beef were stored at 2 ± 1°C in aerobic packaging for 0, 1, 3, and 6 days under retail display conditions. Oxidation stability (thiobarbituric acid‐reactive substances [TBARS]), pH, instrumental color, metmyoglobin formation, and sensory attributes (color and odor) were measured. Omega‐3‐enriched beef increased the oxidation level at day 6 as determined by TBARS (*P *<* *0.05), but the instrumental color was not affected. The enrichment of CLA improved the coordinates of color (*P *<* *0.05) until day 3 and decreased the oxidation at day 6 (*P *<* *0.05). There were no differences in color and odor values among the types of beef during display, except at day 3, when CLA treatments had the highest scores. Addition of GSE decreased the oxidation level (*P *<* *0.001) and did not affect the instrumental color or the sensory parameters.

## Introduction

Consumers are more health‐conscious, driving a trend toward nutritious foods with additional health‐promoting functions. In general, meat and meat products are considered essential in the diet of developed countries (Fernández‐Gines et al. 2005) and its health attributes can be improved by increasing the omega‐3 (n‐3) and conjugated linoleic acid (CLA) fatty acids. The supplementation of ruminant diets with PUFA‐rich lipids is an effective approach to increase the levels of CLA and n‐3 PUFA in meat. However, it is necessary to prevent rumen biohydrogenation of the PUFA in cattle, so that these fatty acids must be supplied in rumen‐protected forms. Therefore, linseed offers a viable alternative as its seed coat may provide some protection to PUFA against rumen biohydrogenation and thus increase the passage of PUFA into the duodenum (Scollan et al. 2001). Moreover, some studies have included rumen‐protected CLA to increase the CLA fatty acids (Gillis et al. 2004; Schlegel et al. 2012).

These polyunsaturated fatty acids influence the meat shelf life due to their propensity to oxidize, leading to the development of rancidity and off‐odor as display time increases (St. Angelo et al. 1990). Moreover, technological operations of meat processing can alter its quality. For instance, ground beef is more susceptible to color deterioration and oxidation than are its whole muscle counterparts (Honikel 2004). Because the color and oxidation stability are very important to retail shelf life, the use of antioxidants is necessary.

The use and applications of natural antioxidants is increasing because studies indicate possible adverse health effects from the use of synthetic antioxidants. Polyphenols are a type of natural antioxidant that, in addition to their antioxidant properties in raw meats (Chen et al. 1999), have specific biological activities that provide beneficial and healthy effects for the human body (Gharras 2009). Grape seed extracts (GSE) are a rich source of polyphenol compounds, especially phenolic acids, flavan‐3‐ols such as catechins and their isomers and proanthocyanidins. The GSE has shown antioxidant activity in beef (Ahn et al. 2002; Bañón et al. 2007; Rojas and Brewer 2007, 2008; Schevey et al. 2013). The antioxidant activity of GSE is dependent on its concentration from 0.02% to 0.1% (Ahn et al. 2002). Gómez et al. (2011) studied several concentrations of GSE in raw beef patties and concluded that 500 mg GSE/kg meat was enough to prevent rancidity of raw beef patties packaged in air and stored for 10 days under retail display conditions.

Therefore, the use of GSE can help to improve the shelf life of ground beef enriched with n‐3 and CLA without affecting oxidative stability or cause adverse effects on sensory characteristics, thus offering a more competitive product on the market. The aim of the present study was to examine the shelf life and oxidative stability in refrigerated raw ground beef enriched with omega‐3 and/or CLA. Grape seed extract was also used as a natural antioxidant to inhibit the lipid oxidation in the ground beef enriched with omega‐3 and/or CLA.

## Material and Methods

### Materials

#### Beef

Beef loin cuts were obtained at 24 h *postmortem* from the right carcass sides of 48 Holstein entire males (10.7 months old) fed with one of four dietary treatments. All animal diets had similar composition but differed in the content of whole linseed and CLA: Control (C, conventional commercial ration, 0% linseed and 0% CLA), omega‐3 (OME3, conventional ration enriched with omega‐3 fatty acids through the addition of 10% linseed), CLA (CLA, conventional ration enriched with CLA through the addition of 2% CLA), and omega‐3 + CLA (OME3 + CLA, conventional ration enriched with omega‐3 and CLA fatty acids through the addition of 10% linseed plus 2% CLA). Animal productive performance and carcass characteristics of these animals were reported by Albertí et al. (2013). Animals were slaughtered with an average live weight of 458.4 ± 16.6 kg at an EU‐licensed commercial abattoir following standard procedures. Vacuum packaged loin cuts were transported to the Public University of Navarre meat laboratory and they were stored at −18°C until required for the experiment (approximately 6 months). The proximate composition and the fatty acid content of loin cuts are shown in Table 1.

**Table 1:** Proximate and fatty acid (FA) composition of ground raw beef enriched with omega‐3 and/or CLA

Beef	C	OME3	CLA	OME3 + CLA
Proximate composition (%)				
Moisture (%)	69.67	69.54	68.61	69.88
Protein (%)	21.66	21.66	21.13	21.65
Fat (%)	5.05	4.86	5.37	4.63
Fatty acid profile (% total fatty acid)				
Saturated fatty acid	42.51	37.84	39.68	38.98
Monounsaturated fatty acid	54.84	58.17	57.48	56.49
Polyunsaturated fatty acid	2.65	3.98	2.84	4.53
Fatty acid content (mg FA/100 g ground beef)				
Omega‐3	1.46	5.87	1.70	6.39
CLA	9.16	7.78	12.56	11.20

Beef from animals fed different diets: C, conventional diet; OME3, omega‐3 polyunsaturated fatty acids (PUFA) supplemented diet; CLA, conjugated linoleic acid supplemented diet; OME3 + CLA, omega‐3 PUFA plus CLA supplemented diet.

#### Extract

A commercial GSE with a polyphenol content of 95% was used. GSE was provided by Exxentia (Madrid, Spain) as a water soluble homogeneous brown powder. The use of GSE (GSE‐0 and GSE‐250, 0 and 250 mg GSE/kg meat, respectively) on ground beef was studied. The selection of the dose used (250 mg GSE/kg meat) was based on a previous study (Gómez et al. 2011).

### Preparation of ground beef

Eight treatments (Table 2) of ground beef were established according to beef enriched with PUFA (C, OME3, CLA, and OME3 + CLA) and the use of GSE (GSE‐0 and GSE‐250): C–GSE‐0, OME3–GSE‐0, CLA–GSE‐0, OME3 + CLA–GSE‐0, C–GSE‐250, OME3–GSE‐250, CLA–GSE‐250 and OME3 + CLA–GSE‐250.

**Table 2:** Experimental design

Treatments	Beef	GSE
C–GSE‐0	C	0
OME3–GSE‐0	OME3	0
CLA–GSE‐0	CLA	0
OME3 + CLA–GSE‐0	OME3 + CLA	0
C–GSE‐250	C	250
OME3–GSE‐250	OME3	250
CLA–GSE‐250	CLA	250
OME3 + CLA–GSE‐250	OME3 + CLA	250

General formulation: 98% ground beef + 2% salt.

Beef from animals fed different diets: C, conventional diet; OME3, omega‐3 polyunsaturated fatty acids (PUFA) supplemented diet; CLA, conjugated linoleic acid supplemented diet; OME3 + CLA, omega‐3 PUFA plus CLA supplemented diet.

GSE dose: GSE‐0, no added GSE; GSE‐250, 250 mg GSE/kg meat.

The frozen beef loin cuts were allowed to thaw for 24 h before being minced. The 12 beef loin cuts from each one of four dietary treatments (C, OME3, CLA, OME3 + CLA) were minced together through a Cato mincer (TALSABELL S.A., Sabadell, Spain). The minced beef (C, OME3, CLA, OME3 + CLA), salt (2%), and GSE (0, 250 mg GSE/kg product) were then blended together by a Sammic mixer (Sammic S.L., Azkoitia, Spain) for 60 sec. The mix was then weighed into portions of 100 g and formed into patties between grease‐proof papers using a patty press, to give average dimensions of 10 cm diameter and 1.5 cm thickness. The meat temperature during processing did not exceed 7°C. The patties were placed in transparent plastic trays covered with a transparent polyvinyl chloride film (PVC) and stored at 2 ± 1°C for 6 days in a retail display cabinet illuminated (10 h/day) with 640 lux of Osram Lumilux Cool White fluorescent lighting, simulating retail display conditions. On each evaluation day (0, 1, 3, and 6), samples were prepared for pH, color, TBARS, and sensory analyses.

### pH values

The pH of the treatments at 0 and 6 days of the display were measured (AOAC 2003) in quadruplicate for each sample. The pH was measured by homogenization in water using a Crison GLP22 pHmeter (Crison Instruments S.A., Barcelona, Spain) equipped with a 6 mm (diameter) penetration probe.

### Color

Color was measured using a Minolta CM‐2002 spectrophotometer (Konica Minolta Business Technologies Inc., Tokyo, Japan) making five measurements per sample. Color parameters were evaluated directly on the patty surface during display (0, 1, 3, and 6 days) using the CIE L*a*b* system, illuminant D65 and 10° as the standard observing point. Results were expressed as CIELab values: Lightness (L*), Redness (a*), Yellowness (b*), Chroma (C*), and Hue angle (H*); C = (a*^2^ + b*^2^)^0.5^; H = arctg b*/a*. The accumulation of metmyoglobin (MMb) on the meat surface was followed by calculating the K/S_572_÷K/S_525_ ratio using the reflectance values, according to Hunt et al. (1991).

### Thiobarbituric acid‐reactive substances

Thiobarbituric acid‐reactive substances (TBARS) values were determined, at 0 and 6 days of the display, in duplicate for each sample, using the method described by Tarladgis et al. (1960). The absorbance was measured at 538 nm in a spectrophotometer (UV‐2101PC; Shimadzu, Kyoto, Japan). The TBARS value, expressed as the mg malonaldehyde/kg meat, was obtained using a conversion factor based on a standard curve using 1,1,3,3‐tetraethoxypropane (TEP).

### Raw ground beef sensory analysis

Quantitative Descriptive Analysis (Stone et al. 1974) was used to assess beef odor and color degradation by a 15‐member trained sensory panel. The methodology carried out was adapted from the one described by Insausti et al. (2001). The panelists consisted of male (*n* = 7) and female (*n* = 8) meat research employees, ranged in age from 22 to 50 years and were familiar with meat and taste panels. They had been trained, in four 30‐min sessions, in evaluating raw ground beef color and odor from retail display using a 15‐cm unstructured line scale. For this, the ground beef samples were placed in transparent plastic trays covered with PVC and stored at 2 ± 1°C for different times up to 10 days in a display cabinet illuminated (10 h/day) to allow the discoloration and the development of off‐odors. Samples were placed at room temperature for 30 min before being presented to the panelists. Training sessions were conducted to familiarize the panelists with the products, attributes to be evaluated, and use of 15‐cm unstructured line scale, and were followed by an open discussion. For rating color degradation, the ground beef samples presenting different color characteristics within the range of the evaluation scale were used (0 = bright red; 7.5 = reddish tan; 15 = tan or brown). For rating odor degradation, the ground beef samples presenting different off‐odor characteristics within the range of the evaluation scale were used (0 = no detectable off‐odor; 7.5 = slight off‐odor; 15 = extreme off‐odor). The acceptability limit for odor and color was anchored in the middle of the line (7.5 cm from each end).

Evaluation occurred on days 0, 1, 3, and 6. The raw ground beef samples were placed at room temperature for 30 min before being presented to the panelists. The samples were taken as needed from the display cabinet and identified with 3‐digit random numbers. Each panelist received samples of each treatment randomly numbered and served. Sessions were carried out in individual booths. Firstly, odor evaluations were performed under soft red light (≈100 lux). The odor of raw ground beef was evaluated by the panelists just after opening each pack and the result was marked on a paper scorecard prepared for each of the panelists. They rated for the attribute raw beef odor using a 15‐cm unstructured line scale. Then, color evaluations were evaluated under white light (≈450 lux) by the panelists using a 15‐cm unstructured line scale. The results were quantified by measuring the distance in centimeters of the panelists' mark from the left side.

### Cooked ground beef sensory analysis

Quantitative Descriptive Analysis (Stone et al. 1974) was used to assess beef odor and color of cooked ground beef by the same 15‐member trained sensory panel used to evaluate the raw ground beef. The panelists had been trained, in four 30‐min sessions, in evaluating cooked ground beef color and odor from display using a 15‐cm unstructured line scale. The ground beef samples used for training were treated in the same conditions of packaging, temperature, illumination, and time as those used for the training on raw ground beef. The samples were cooked in a double hot‐plate grill preheated to 200°C until the internal temperature reached 71°C, using individual thermocouples inserted into the geometric center of the meat. The cooked samples were stored at 55°C in a heater until their evaluation. Training sessions were conducted to familiarize the panelists with the products, attributes to be evaluated, and use of 15‐cm unstructured line scale, and were followed by an open discussion. For rating color, the cooked ground beef samples presenting different color characteristics within the range of the evaluation scale were used (0 = pale pink; 7.5 = pink‐tan; 15 = brown). For rating odor, cooked ground beef samples presenting different odor characteristics within the range of the evaluation scale were used (0 = no warmed over flavor, WOF; 7.5 = slight WOF; 15 = extremely strong WOF). The acceptability limit for odor and color was anchored in the middle of the line (7.5 cm from each end).

The samples were taken from each of the treatments at 2 days of the display. They were cooked in a double hot plate grill preheated to 200°C until the internal temperature reached 71°C, using individual thermocouples inserted into the geometric center of the meat. They were coded and kept warm in a heater for between 5 and 15 min until sensory analyses. The methodology carried out to assess cooked beef odor and color degradation was the one described previously for raw ground beef. The acceptability limit for odor and color was anchored in the middle of the line (7.5 cm from each end).

### Statistics

Data were analyzed using the general linear model (GLM) procedure (IBM‐SPSS version 21 for Windows, SPSS Inc, Chicago, IL, USA). Duplicate batches of sample patties were prepared for all treatments and the effect of replication was not significant. For pH, TBARS index, instrumental color readings, and the sensory analysis data for the raw ground beef, the statistical model included the fixed effects of beef type (B), GSE and display time (T) as well as the interactions among them, and the residual error. For the sensory analysis data for the cooked ground beef, the statistical model included the fixed effects of B and GSE as well as the interactions between them and the residual error. Differences among means were analyzed by Tukey's test. Pearson's correlation coefficients between variables were calculated. The level of significance was set at *P *<* *0.05 in all cases. Multivariate analysis, namely, factor analysis, was used to examine the relationships among all the variables considered. Factors were extracted using the principal component analysis (PCA). Varimax rotation was applied to the factors to facilitate interpretation and maximize the explained variance.

## Results and Discussion

### pH

Table 3 shows the pH values of the treatments, whose triple interaction BxGSExT was not significant (*P *>* *0.05). The interaction BxGSE and the effect of B were statistically significant (*P *<* *0.05) at 0 day, whereas only the treatment had significant effect (*P *<* *0.05) on pH values at 6 day. The omega‐3‐enriched beef had lower pH values than those of control beef (5.25 vs. 5.32; *P *<* *0.05). However, in previous studies there were no differences on pH of beef from bulls fed linseed (Juárez et al. 2012). The pH values were not affected by time in the raw ground beef, except the OME3 + CLA treatment that had lower pH values at 6 day. GSE supplementation had no significant effect on pH during the display; likewise, Bañón et al. (2007) and Rojas and Brewer (2007) also found no differences when GSE was added in ground beef.

**Table 3:** Changes in pH values in raw ground beef enriched with omega‐3 and/or CLA stored in aerobic packaging for 0 and 6 days under retail display conditions

GSE	GSE‐0				GSE‐250				SEM	*P*‐value		
Beef	C	OME3	CLA	OME3 + CLA	C	OME3	CLA	OME3 + CLA		B	GSE	BxGSE
Day												
* *0	5.33^a^	5.26^bc^	5.29^abc^	5.30^ab^	5.32^a^	5.27^abc^	5.31^ab^	5.24^c^	0.01	<0.001	0.430	0.017
* *6	5.32	5.23	5.18	5.17	5.30	5.22	5.17	5.15	0.06	0.045	0.405	0.823
* *SEM	0.03	0.07	0.05	0.04	0.02	0.02	0.05	0.03				
* P*‐value	0.863	0.498	0.188	0.048	0.487	0.071	0.114	0.131				

Mean values with different superscripts in the same row (different dietary treatments on the same day of storage) were significantly different (*P *<* *0.05).

SEM, Standard error of mean; B, fixed effect of beef type; GSE, fixed effect of added grape seed extract.

GSE dose: GSE‐0, no added GSE; GSE‐250, 250 mg GSE/kg meat.

Beef from animals fed different diets: C, conventional diet; OME3, omega‐3 polyunsaturated fatty acids (PUFA) supplemented diet; CLA, conjugated linoleic acid supplemented diet; OME3 + CLA, omega‐3 PUFA plus CLA supplemented diet.

### Thiobarbituric acid‐reactive substances

Table 4 shows the level of lipid oxidation of the treatments. There was a significant interaction BxGSExT (*P *<* *0.001) for TBARS of ground beef during display. The beef type and GSE factors as well as the interaction of BxGSE had no significant effects (*P *>* *0.05) on lipid oxidation at day 0. Nevertheless, the interaction of BxGSE and beef type and GSE factors was statistically significant (*P *<* *0.001) at day 6. TBARS values of treatments without GSE increased gradually from 0.57 mg MDA/kg to around 3.24 mg MDA/kg (*P *<* *0.001) during display, whereas treatments with GSE had constant TBARS values (around 0.53 mg MDA/kg; *P *>* *0.05) and lower than the value of 2 mg MDA/kg, which is the upper limit of rancidity for the acceptability of beef consumers (Campo et al. 2006).

**Table 4:** Changes in thiobarbituric acid reagent substances (TBARS, mg MDA/kg meat) in raw ground beef enriched with omega‐3 and/or CLA stored in aerobic packaging for 0 and 6 days under retail display conditions

GSE	GSE‐0				GSE‐250				SEM	*P*‐value		
Beef	C	OME3	CLA	OME3 + CLA	C	OME3	CLA	OME3 + CLA		B	GSE	BxGSE
Day												
0	0.51	0.62	0.44	0.72	0.49	0.49	0.36	0.63	0.13	0.241	0.400	0.982
6	2.38^b^	4.59^a^	1.56^c^	4.42^a^	0.47^d^	0.61^d^	0.48^d^	0.70^d^	0.16	<0.001	<0.001	<0.001
SEM	0.10	0.15	0.25	0.08	0.12	0.17	0.12	0.09				
*P*‐value	<0.001	<0.001	0.018	<0.001	0.928	0.650	0.483	0.584				

Mean values with different superscripts in the same row (different dietary treatments on the same day of storage) were significantly different (*P *<* *0.05).

SEM, Standard error of mean; B, fixed effect of beef type; GSE, fixed effect of added grape seed extract.

GSE dose: GSE‐0, no added GSE; GSE‐250, 250 mg GSE/kg meat.

Beef from animals fed different diets: C, conventional diet; OME3, omega‐3 polyunsaturated fatty acids (PUFA) supplemented diet; CLA, conjugated linoleic acid supplemented diet; OME3 + CLA, omega‐3 PUFA plus CLA supplemented diet.

The treatments without GSE that suffered the highest oxidation level were those which used beef enriched with omega‐3 (4.51 mg MDA/kg) because polyunsaturated fatty acids are more susceptible to lipid oxidation and decrease the lipid stability during refrigerated storage. Previous studies reported that steaks with higher omega‐3 fatty acid (FA) content (from flaxseed diets) showed less lipid stability during retail display (Juárez et al. 2012). Moreover, CLA–GSE‐0 treatment showed the lowest TBARS values within treatments without GSE. Ha et al. (1990) suggested that CLA may have an antioxidant effect, whereas Fagali and Catalá (2008) and Yu (2001) demonstrated that CLA can provide immediate protection against free radicals, which would protect against lipid oxidation. Dietary CLA reduced TBARS levels and lipid oxidation of pork loin (Joo et al. 2002). Furthermore, direct addition of CLA during the preparation of beef patties decreased TBARS production during refrigerated storage (Chae et al. 2004; Hur et al. 2004). These findings could demonstrate that CLA reduces the formation of fatty acid free radicals and subsequent oxidation reactions.

In contrast with treatments without GSE that were affected by display time (*P *<* *0.05), GSE treatments were not influenced over time (*P *>* *0.05) because of the antioxidant action of GSE, that can delay the formation of TBARS. These findings are in agreement with previous studies in raw and cooked beef (Ahn and others 2004; Ahn et al. 2007; Bañón et al. 2007; Rojas and Brewer 2007). The antioxidant activity of GSE has been associated with the presence of phenolic compounds (Cuppett 2001), whose main mechanism is by acting as free radical scavengers. In the present study, we found that using 250 mg GSE/kg meat, with 95% of polyphenols, had an antioxidant effect in ground beef enriched with omega‐3 and/or CLA and packaged in air for 6 days under retail display conditions. Ahn et al. (2002) reported that antioxidant activity of GSE was dependent on the concentration from 0.02% to 0.1% in cooked ground beef. GSE at concentrations as low as 0.1% reduced secondary oxidation products in beef during refrigerated storage (Ahn et al. 2007). Rojas and Brewer (2008) compared the antioxidant effect of GSE in beef and pork and concluded that the doses of GSE 0.01% and 0.02% were effective in both meat species. Furthermore, GSE has also been effective as lipid antioxidant in meat such as pork (Lorenzo et al. 2014) or chicken (Brannan 2008).

### Color

Table 5 shows the effects of beef type, GSE, and display time on color parameters (L*, a*, b*, C*, and H*) of raw ground beef in aerobic packaging for 6 days under retail display conditions. Although the triple interaction of BxGSExT was not significant (*P *>* *0.05), table analysis of it provides valuable information.

**Table 5:** Changes in Lightness (L*), Redness (a*), Yellowness (b*), Chroma (C*), and Hue angle (H*) in the raw ground beef enriched with omega‐3 and/or CLA stored in aerobic packaging for 0, 1, 3, and 6 days under retail display conditions

GSE	GSE‐0				GSE‐250				SEM	*P*‐value		
Beef	C	OME3	CLA	OME3 + CLA	C	OME3	CLA	OME3 + CLA		B	GSE	BxGSE
Day												
L*												
0	39.11^1^	39.51	39.82	38.13^12^	38.66^1^	39.84	40.02^12^	41.13^1^	0.82	0.626	0.190	0.171
1	36.25^12^	36.89	38.11	37.41^12^	37.87^12^	38.95	40.57^1^	40.20^1^	0.63	0.003	<0.001	0.813
3	33.65^2^	36.50	38.90	36.47^2^	34.29^3^	37.90	37.48^2^	36.38^2^	0.68	<0.001	0.786	0.207
6	37.57^bc1^	38.30^bc^	39.27^ab^	39.62^ab1^	36.08^c23^	39.00^abc^	41.61^a1^	42.07^a1^	0.70	<0.001	0.049	0.022
SEM		0.81	0.86	0.58	0.74	0.67	0.55	0.79	0.64			
*P*‐value	<0.001	0.069	0.221	0.034	<0.001	0.122	0.006	<0.001				
a*												
0	14.23^1^	15.30^1^	15.66^1^	13.94^1^	13.31^1^	15.13^1^	15.81^1^	15.29^1^	0.74	0.061	0.847	0.494
1	8.57^2^	10.12^2^	10.49^2^	9.17^2^	9.72^2^	11.71^2^	12.35^2^	11.43^2^	0.41	<0.001	<0.001	0.597
3	6.70^2^	8.86^2^	9.66^23^	8.81^2^	7.10^3^	7.89^3^	10.65^2^	10.07^23^	0.44	<0.001	0.201	0.065
6	8.30^ab2^	6.49^d3^	7.98^abcd3^	7.72^abcd2^	6.74 ^cd3^	8.02^abc3^	6.93^bcd3^	8.58^a3^	0.35	0.067	0.830	<0.001
SEM		0.54	0.51	0.59	0.46	0.42	0.52	0.53	0.49			
*P*‐value	<0.001	<0.001	<0.001	<0.001	<0.001	<0.001	<0.001	<0.001				
b*												
0	10.27^1^	10.50^1^	11.91^1^	10.68^1^	9.36^1^	11.17^1^	12.34^1^	12.16^1^	0.51	<0.001	0.248	0.133
1	7.38^c2^	7.76^c2^	8.98^abc2^	7.78^c2^	7.61^c2^	8.31^bc2^	9.94^ab2^	10.30^a2^	0.38	<0.001	<0.001	0.018
3	8.11^abc2^	7.45^bc2^	9.51^a2^	7.80^abc2^	6.56^c2^	6.51^c3^	9.20^ab2^	9.34^ab2^	0.44	<0.001	0.315	0.005
6	8.52^a2^	7.15^ab2^	8.45^a2^	6.66^b2^	6.90^ab2^	7.87^ab23^	7.30^ab3^	7.43^ab3^	0.39	0.357	0.393	0.001
SEM	0.39	0.53	0.46	0.48	0.39	0.46	0.40	0.33				
*P*‐value	<0.001	<0.001	<0.001	<0.001	<0.001	<0.001	<0.001	<0.001				
C*												
0	17.65^1^	18.58^1^	19.79^1^	17.70^1^	16.34^1^	18.84^1^	20.14^1^	19.58^1^	0.70	0.001	0.554	0.170
1	11.33^2^	12.78^2^	13.86^2^	12.05^2^	12.39^2^	14.36^2^	15.86^2^	15.40^2^	0.50	<0.001	<0.001	0.128
3	10.59^c2^	11.60^bc23^	13.70^ab2^	11.79^bc2^	9.70^c3^	10.29^c3^	14.12^a2^	13.78^ab2^	0.51	<0.001	0.892	0.008
6	11.93^a2^	9.74^c3^	11.69^ab3^	10.20^bc2^	9.71^c3^	11.25^abc3^	10.18^bc3^	11.65^ab3^	0.36	0.584	0.441	<0.001
SEM	0.55	0.65	0.48	0.52	0.43	0.62	0.49	0.46				
*P*‐value	<0.001	<0.001	<0.001	<0.001	<0.001	<0.001	<0.001	<0.001				
H*												
0	36.22^3^	34.40^2^	37.52^2^	37.62	35.19^3^	36.46^2^	38.17^2^	38.68	1.76	0.285	0.583	0.849
1	40.91^23^	37.14^2^	40.76^12^	40.12	37.99^23^	35.43^2^	38.76^2^	41.94	1.17	0.001	0.152	0.205
3	50.17^a1^	39.67^b2^	44.62^ab12^	41.20^b^	42.72^ab12^	39.06^b2^	40.91^b12^	43.32^ab^	1.70	0.001	0.048	0.040
6	45.49^12^	47.53^1^	46.54^1^	40.58	45.65^1^	44.39^1^	46.60^1^	42.07	2.04	0.049	0.805	0.707
SEM	1.55	1.61	2.23	1.53	1.74	1.32	1.89	1.55				
*P*‐value	<0.001	<0.001	0.033	0.381	0.001	<0.001	0.013	0.199				

Mean values with different superscripts in the same row (different dietary treatments on the same day of storage) were significantly different (*P *<* *0.05).

SEM, Standard error of mean; B, fixed effect of beef type; GSE, fixed effect of added grape seed extract.

GSE dose: GSE‐0, no added GSE; GSE‐250, 250 mg GSE/kg meat.

Beef from animals fed different diets: C, conventional diet; OME3, omega‐3 polyunsaturated fatty acids (PUFA) supplemented diet; CLA, conjugated linoleic acid supplemented diet; OME3 + CLA, omega‐3 PUFA plus CLA supplemented diet.

L* double interactions B×T (*P *=* *0.006), GSE×T (*P *=* *0.029), and BxGSE (*P *=* *0.057) were analyzed. There were significant differences (*P *<* *0.01) on L* values from day 1 according to the beef type used, showing C treatments the lowest L* values. CLA treatments had L* values higher than those of C treatments. Hur et al. (2004) reported that CLA addition (0.5 or 2%) increased L* compared to control patties on day 7 in raw refrigerated beef patties. In general, the mean L* value for the control treatments was lower than those of OME3 and OME3 + CLA treatments. Consequently, beef enriched with omega‐3 enhanced the ground beef's lightness, which is in agreement with a report by Juárez et al. (2012). Regarding the time effect, L* values slightly decreased over time (*P *<* *0.001) in control ground beef, whereas in the other treatments L* values in 0 day were similar than those of 6 day (Table 5). No clear data trends were observed by GSE addition, these results are in agreement with those reported by other authors in pork patties (Lorenzo et al. 2014).

The interactions BxGSE, BxT, and GSE×T were statistically significant (*P *<* *0.05) for a* values. The a* values presented significant differences among treatments according to the beef type used. The control beef showed the lowest a* values until day 3. CLA addition resulted in a* values higher than those of control treatments (*P *<* *0.05) until day 3 of refrigerated storage. The addition of 0.5 and 2% of CLA increased a* values in beef patties (Hur et al. 2004), whereas a* was not altered when either 2 or 4% of CLA was added directly into beef patties (Chae et al. 2004). Likewise, omega‐3 treatments had a* values higher than those of control treatments (*P *<* *0.05) until day 3. All treatments showed a significant (*P *<* *0.001) decrease in redness (around 49%) due to the oxidation of pigments during refrigeration of meat products. GSE addition had a significant effect (*P *<* *0.05) on a* of ground beef at day 1, resulting in higher values for a* in the GSE treatments. GSE addition (0.01 and 0.02%) did not change measures of redness in beef patties (Rojas and Brewer 2007).

The b* significant interactions (*P *<* *0.01) of B×T, GSE×T, and BxGSE were studied. There were significant differences (*P *<* *0.01) on b* values according to the beef type used. The mean b* values of CLA treatments were higher than those of control treatments (*P *<* *0.05) to day 3 of display. However, when 2 or 4% of CLA was added directly into beef patties, the yellowness was not altered (Chae et al. 2004). In general, the b* values in omega‐3‐enriched beef were similar to those of the control beef. The yellowness was mainly affected by display time (*P *<* *0.001) and the b* values decreased of by around 31% in the treatments. In general, the b* values were not influenced by the addition of GSE (*P *>* *0.05), similar to results found in other studies (Rojas and Brewer 2007).

The interactions of BxGSE, BxT, and GSE×T were statistically significant (*P *<* *0.001) for C*.While, only the BxT interaction was significant for H* (*P *=* *0.001). There were no clear differences among treatments for C* and H* values during the display. In general, there was a decrease in C* (*P *<* *0.001), while H* increased (*P *<* *0.05) over time. These color changes are normally associated with the loss of redness (Bañón et al. 2007) and the loss of stability of the color in meat that can result in undesirable color for the consumer. In general, C* and H* were not influenced by the addition of 250 mg GSE/kg meat (*P *>* *0.05) during display of the ground beef packaged in air. However, differences for C*and H* were found in beef patties with 100 SO_2_ + 300 GSE (mg/kg meat) compared to patties without additives (Bañón et al. 2007).

**Figure 1:**
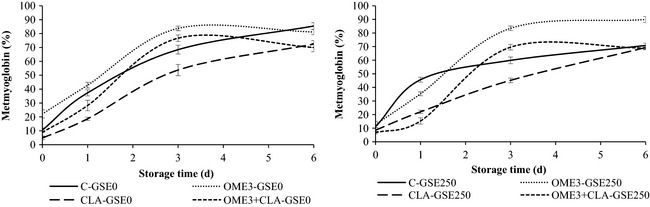
Changes in the percentage of surface metmyoglobin (means ± SE) in the raw ground beef enriched with omega‐3 and/or CLA stored in aerobic packaging for 0, 1, 3, and 6 days under retail display conditions. Beef from animals fed different diets: C, conventional; OME3, omega‐3; CLA, conjugated linoleic acid; OME3 + CLA, omega‐3 plus CLA. GSE dose: GSE‐0, no added GSE; GSE‐250, 250 mg GSE/kg meat.

The relative percentages of MMb measured at the surface of the ground beef during 6 days of display are shown in Figure 1. There was a significant BxGSExT interaction (*P *<* *0.001) for percentage of MMb of raw ground beef during display. There were significant differences (*P *<* *0.001) in MMb percentages according to the beef type used. The CLA treatments had the lowest % MMb to day 3 of the display. CLA sources for fat improved the oxymyoglobin stability due to the antioxidant effect of CLA (Hur et al. 2004). In contrast, the OME treatments had the highest MMb percentages because the color change is partially due to increased lipid oxidation associated with unsaturated fatty acids. Also, there are other factors such as grinding, light, and salt which promote the oxidation of pigments and for that, in all treatments MMb gradually increased during the 6 days of display (*P *<* *0.001). In addition, in the present study the samples were in aerobic packs (PVC) which promoted the exposure of myoglobin to O_2_ and the development of aerobic microorganisms. This may be one of the reasons leading to discoloration of beef. Lavieri and Williams (2014), despite using three types of packaging (vacuum, PVC, and MAP) in ground beef, observed an increase in psychrotrophic bacteria counts which led to discoloration in the ground beef during refrigerated storage. GSE addition had a significant effect (*P *<* *0.01) on % MMb of ground beef at day 3, resulting in lower values for % MMb in the GSE treatments, except in the OME3 treatment where the GSE was not able to decrease the MMb. Furthermore, GSE decreased the % MMb in control beef at day 6 (85.45 vs. 70.81), resulting in similar values to those of CLA‐enriched beef (CLA and OME3 + CLA treatments with or without GSE, 70.13%) for that day. GSE was not able to delay the color deterioration in the OME3‐enriched beef over time, and the OME3 treatments had 85.51% of MMb at day 6. Therefore, in ground beef it would be necessary to apply the hurdle technology by using natural antioxidants such as GSE, as well as additives and modified atmosphere or vacuum packaging, to protect fully these meat products from the discoloration and microbial growth. For instance, Bañón et al. (2007) reported differences in MMb in beef patties with 100 SO_2_ + 300 GSE (mg/kg meat) compared to control beef (without additives), because the sulfite delayed the color deterioration. Moreover, the combined use of antioxidants and modified atmosphere packaging for meat increases the shelf life of fresh meat (Sánchez‐Escalante et al. 2003; Lorenzo et al. 2014).

It should be noted that differences in the methods used to enrich the beef with omega‐3 and CLA, grinding, salt, color of GSE, different ratios of lean/fat, light, display time, and packaging system used, make it difficult to compare these results to those obtained by other authors. For instance, as result of omega‐3 and CLA supplementation in the diet compared to omega‐3 and CLA directly added to meat, these fatty acids are located at different positions within the meat matrix and some functions could be different. The grinding of meat destroys the aerobic system which may partially explain the accelerated oxidation of the pigment in the ground beef compared to the whole muscle (Honikel 2004). Salt promotes lipid oxidation in raw and cooked meat and accelerates metmyoglobin formation and discoloration in raw meat (Rhee 1999).

### Sensory analysis

#### Raw ground beef sensory analysis

**Figure 2:**
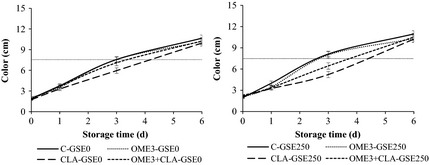
Sensory evaluation: color of the raw ground beef enriched with omega‐3 and/or CLA stored in aerobic packaging for 0, 1, 3, and 6 days under retail display conditions (15 cm: maximal discoloration scores; 7.5 cm: acceptability limit). Beef from animals fed different diets: C, conventional diet; OME3, omega‐3 polyunsaturated fatty acids (PUFA) supplemented diet; CLA, conjugated linoleic acid supplemented diet; OME3 + CLA, omega‐3 PUFA plus CLA supplemented diet. GSE dose: GSE‐0, no added GSE; GSE‐250, 250 mg GSE/kg meat.

**Figure 3:**
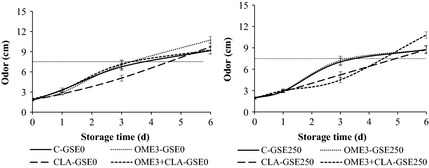
Sensory evaluation: odor of the raw ground beef enriched with omega‐3 and/or CLA stored in aerobic packaging for 0, 1, 3, and 6 days under retail display conditions (15 cm: maximal odor scores; 7.5 cm: acceptability limit). Beef from animals fed different diets: C, conventional diet; OME3, omega‐3 polyunsaturated fatty acids (PUFA) supplemented diet; CLA, conjugated linoleic acid supplemented diet; OME3 + CLA, omega‐3 PUFA plus CLA supplemented diet. GSE dose: GSE‐0, no added GSE; GSE‐250, 250 mg GSE/kg meat.

The evolution of color and odor of the raw ground beef in aerobic packaging for 6 days under retail display conditions is shown in Figures 2 and 3, respectively. There was a significant interaction BxGSExT (*P *<* *0.001) for odor, but not for color (*P *>* *0.05).

Beef type had no significant effect on color during display, except at day 3 (Fig. 2). On that day, the ground beef enriched with CLA had better scores for color than the other treatments (5.59 vs. 7.47), which could mean that CLA had a positive effect on the color and would support the instrumental color results (Table 5). Moreover, the scores for color in ground beef enriched with omega‐3 were similar (*P *>* *0.05) to those of control beef during the 6 days of display. Color values of treatments increased gradually during storage from 1.99 to 10.38 (*P *<* *0.001). These results correspond to the discoloration of the beef which is due to lipid oxidation and the oxidation of pigments, as has been previously explained in instrumental color results. The GSE did not significantly influence the color except at day 0, resulting in higher values in the GSE treatments (1.88 vs. 2.09, *P *<* *0.05). Furthermore, it should be noted that GSE addition improved the color value of OME3 + CLA treatment at day 3. Rojas and Brewer (2008) did not find differences for visual color when 0.02% of GSE was added to beef that was frozen during 4 months.

Similar to the sensory color, odor was not affected by beef type during display, except at day 3 (Fig. 3), when CLA improved the odor values compared to the other beef (5.14 vs. 7.04, *P *<* *0.001). These results could mean that CLA had a positive effect on the odor at day 3. Moreover, omega‐3 addition did not affect the odor values of ground beef during the 6 days. Odor values of treatments increased over time from 1.90 to 9.49 (*P *<* *0.001). The development of off‐odors might be explained by the secondary products of the lipid oxidation that happens during refrigerated storage (Jongberg et al. 2011) and the spoilage of the beef due to microbial populations which lead to the formation of microbial slime formation, off‐odor, and discoloration (Lavieri and Williams 2014). It should be noted that no slime formation was observed on the surface of the ground beef in the present study. GSE addition improved the odor value of OME3 + CLA treatment at day 3 (7.15 vs. 4.51, *P *<* *0.001) and that of OME3 treatment at day 6 (10.74 vs. 8.77, *P *<* *0.01). Rojas and Brewer 2008 reported that the addition of 0.02% GSE in beef did not affect odor described as raw meat, grassy, herbal, acid, and sweaty.

In the present study, odor and color deteriorated similarly over the time of display. The scores in both sensory parameters were higher than the acceptability value (7.5 cm) from day 3, so that the shelf life would be limited to 3 days for these raw ground beef packaged in PVC and stored under retail display conditions, results which support those obtained by Lavieri and Williams (2014).

#### Cooked ground beef sensory analysis

No significant interaction of BxGSE was found (*P *>* *0.05; data not shown) either sensory parameter. All treatments had color and odor values lower than the acceptability value (7.5 cm), so all of them were sensorially acceptable.

Beef type had significant effect on color (*P *<* *0.05). Control treatments were the best evaluated, as well as the OME3 + CLA treatments. However, individual enrichment of omega‐3 or CLA in cooked beef did not improve the scores of color compared to control beef. GSE addition did not significantly affect the color of cooked ground beef. These results are in agreement with those reported in other studies about cooked beef during refrigerated storage (Bañón et al. 2007; Jongberg et al. 2011).

Polyunsaturated fatty acid supplementation significantly affected odor (*P *=* *0.016) of cooked ground beef. Control treatments were the best evaluated compared to the other treatments. In the present study, WOF was not detected in cooked ground beef at day 2, although commonly they are developed within 1–3 days of refrigerated storage. The odor parameter in GSE treatments was better evaluated than in treatments without GSE (*P *=* *0.003). Rojas and Brewer (2007) reported that beef patties with 0.02% of GSE had better scores for wet cardboard and rancidity parameters compared to control patties. These findings might justify that GSE can have potential to control some of the negative sensory characteristics associated with unpleasant flavors. However, Bañón et al. (2007) did not find significant changes in the odor of cooked beef patties with GSE and low concentrations of sulfite. The different results among the authors could be explained by the presence of additives, packaging type, lipid composition of beef and the content of polyphenolic compounds of GSE used in each of the studies.

### Principal components analysis

Table 6 shows the correlation coefficients among the variables studied in the raw ground beef (TBARS, pH, L*, a*, b*, C*, H*, % MMb, color, and odor). The color coordinates a*, b*, and C* were positively correlated with each other, and negatively correlated with H* and % MMb. L* was only negatively correlated with the pH. Moreover, color and odor had high correlation coefficient with each other. Likewise, the correlation between a* and b* with sensory parameters was negative and high. TBARS was negatively correlated with a*, b*, and C*, and positively correlated with H*, % MMb, and odor.

**Table 6:** Pearson's correlation coefficient among response variables: TBARS, pH, L*, a*, b*, C*, H*, % MMb, color and odor

	TBARS	pH	L^a^	a^a^	b^a^	C^a^	H^a^	% MMb	Odor	Color
TBARS	–									
PH	−0.051	–								
L^a^	−0.204	−0**.635** b	–							
a^a^	−0**.528** ^*^	0.401	0.207	–						
b^a^	−0**.520** a	0.406	0.205	**0.952** b	–					
C^a^	−0**.535** a	0.405	0.214	**0.996** b	**0.976** b	–				
H^a^	**0.426**	−0.300	−0.173	−0**.900** b	−0**.730** b	−0**.857** b	–			
% MMb	**0.520** a	−0.414	−0.182	−0**.956** b	−0**.887** b	−0**.946** b	**0.887** b	–		
Odor	**0.554** a	−0.485	−0.072	−0**.964** b	−0**.895** b	−0**.951** b	**0.897** b	**0.966** b	–	
Color	0.496	−0.475	−0.130	−**0.975** b	−0**.903** b	−0**.963** b	**0.905** b	**0.980** b	**0.986** b	–

TBARS, thiobarbituric acid‐reactive substances.

a Significance at level *P *<* *0.05.

b Significance at level *P *<* *0.01.

Principal component analysis (PCA) showed that about 94.30% of the variability was explained by the three main principal components, and 70.94% of it was accounted for by the principal component 1 (PC1). The increase in metmyoglobin, H*, color, and odor and the decrease in a*, b*, and C* clearly reflect a degradation in ground beef quality; hence, PC1 was a ground beef quality degradation factor. The principal component 2 (PC2, 16.65%) was formed by L* and pH. Principal component 3 (PC3, 6.71%) was formed by TBARS, so the PC3 was a ground beef lipid oxidation factor.

**Figure 4:**
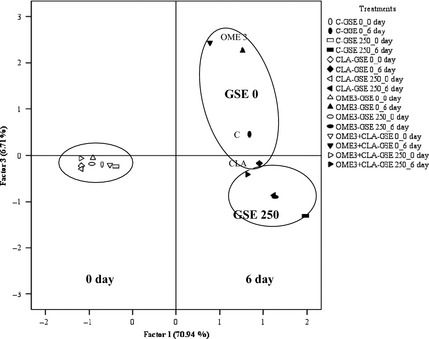
Plot of treatments of raw ground beef for 0 and 6 days on the bidimensional space formed by factors 1 and 3 obtained by principal component analysis of TBARS, pH, L*, a*, b*, C*, H*, % MMb, color, and odor variables. Beef from animals fed different diets: C, conventional diet; OME3, omega‐3 polyunsaturated fatty acids (PUFA) supplemented diet; CLA, conjugated linoleic acid supplemented diet; OME3 + CLA, omega‐3 PUFA plus CLA supplemented diet. GSE dose: GSE‐0, no added GSE; GSE‐250, 250 mg GSE/kg meat.

When plotting the treatments of raw ground beef for 0 and 6 days on the same bidimensional space, a clear separation was observed by PC1 (Fig. 4). Treatments at day 0 were placed on the left side, whereas treatments at day 6 were placed on the right side. Consequently, the variables H*, % MMb, color, and odor increased with increasing display time, whereas a*, b*, and C* decreased, so they can be used as indicators of the loss of raw ground beef quality. Likewise, Insausti et al. (2008) reported a clear separation by factor 1, related to the beef quality degradation, when plotting the days of storage on the same bidimensional space.

Moreover at day 6, PC3 separated GSE treatments (negative side of PC3) from the treatments without GSE (positive side of PC3). The PC3 was related to TBARS, so the lipid oxidation can be used as indicator of the effectiveness of GSE in raw ground beef at day 6 of display. The PCA also separated sausages with GSE from the control group (without antioxidants) by factor 1, which was positively related to moisture content, aw, color parameters, and acetic concentration and inversely related to TBARS and TPA (Lorenzo et al. 2013). In the present study, it should be noted that CLA treatment without GSE (CLA–GSE‐0) was placed at the negative side of PC3 because CLA had an antioxidant effect, such as was explained in “TBARS” section. Likewise, OME3 treatments were at the top of the positive side of PC3 as they presented the highest level of oxidation due to their enrichment with omega‐3 fatty acids. Therefore, the PC3 could differentiate the ground beef without added GSE according the enrichment or no with omega‐3 and/or CLA, because PC3 was related with the oxidative stability which depended on the lipid composition of the ground beef.

## Conclusions

The enrichment of beef with omega‐3 and CLA improves the lipid profile of the beef, although the oxidative stability is impaired. The enrichment of omega‐3 and omega‐3 plus CLA by modifying the diet of bulls have not been enough to cause variations in the instrumental color or the sensory parameters, so, the visual appearance of enriched beef is similar to conventional beef. The results pointed to the potential value of CLA enrichment to stabilize the lipid oxidation. Furthermore, the color in beef enriched with CLA was improved until day 3, which shows potential to produce more attractive products for the consumers. According to the sensory analyses, the shelf life of the ground enriched beef would be 3 days, under aerobic packaging and retail display conditions.

GSE addition prevented rancidity in ground raw ground beef enriched with omega‐3 and/or CLA and did not affect the instrumental color or the sensory parameters in ground beef. The results suggest that GSE can be a technologically viable alternative for stabilizing the lipid oxidation in new fresh meat products, although it should be used in conjunction hurdle technologies to reduce the discoloration and the microbial growth.

## Conflict of Interest

None declared.
